# Cibinetide Protects Isolated Human Islets in a Stressful Environment and Improves Engraftment in the Perspective of Intra Portal Islet Transplantation

**DOI:** 10.1177/09636897211039739

**Published:** 2021-09-09

**Authors:** Ming Yao, Anna Domogatskaya, Nils Ågren1, Masaaki Watanabe, Kazuaki Tokodai, Michael Brines, Anthony Cerami, Bo-Göran Ericzon, Makiko Kumagai-Braesch, Torbjörn Lundgren

**Affiliations:** 1 Division of Transplantation Surgery, CLINTEC, Karolinska Institutet, and Department of Transplantation Surgery, Karolinska University Hospital Huddinge, 141 86 Stockholm, Sweden; 2 Araim Pharmaceuticals, Tarrytown, NY 105 91, USA

**Keywords:** Islet Transplantation, Immunosupression, Diabetes, Inflammation, Insulin

## Abstract

During intra-portal pancreatic islet transplantation (PITx), innate immune reactions such as the instant blood mediated inflammatory reaction (IBMIR) cause an immediate loss of islets. The non-hematopoietic erythropoietin analogue cibinetide has previously shown islet-protective effects in mouse PITx. Herein, we aimed to confirm cibinetide’s efficacy on human islets, and to characterize its effect on IBMIR. We cultured human islets with pro-inflammatory cytokines for 18 hours with or without cibinetide. ATP content and caspase 3/7 activity were measured. Dynamic glucose perfusion assay was used to evaluate islet function. To evaluate cibinetides effect on IBMIR, human islets were incubated in heparinized polyvinyl chloride tubing system with ABO compatible blood and rotated for 60 minutes to mimic the portal vein system. Moreover, human islets were transplanted into athymic mice livers via the portal vein with or without perioperative cibinetide treatment. The mice were sacrificed six days following transplantation and the livers were analyzed for human insulin and serum for human C-peptide levels. Histological examination of recipient livers to evaluate islet graft infiltration by CD11b^+^ cells was performed. Our results show that cibinetide maintained human islet ATP levels and reduced the caspase 3/7 activity during culture with pro-inflammatory cytokines and improved their insulin secreting capacity. In the PVC loop system, administration of cibinetide reduced the IBMIR-induced platelet consumption. In human islet to athymic mice PITx, cibinetide treatment showed an increased amount of human insulin in the livers and higher serum human C-peptide, while histological examination of the livers showed reduced infiltration of pro-inflammatory CD11b^+^ cells around islets grafts compared to the controls. In summary, Cibinetide protected isolated human islets in a pro-inflammatory milieu and reduced IBMIR related platelet consumption. It improved engraftment of human islets in athymic mice. The study confirms that cibinetide is a promising agent to be used in clinical PITx.

## Background

Intra-portal pancreatic islet transplantation (PITx) offers a low invasive treatment option for type 1 diabetics with life quality reducing hypoglycemic events. The outcomes after PITx have improved after the implementation of “The Edmonton protocol,” and the long-term results of insulin independence have reached over 50% in high volume PITx-centers^1^. Even if patients do not achieve long lasting insulin independence, it has been shown that the presence of a maintained C-peptide can stabilize or even improve secondary diabetic complications and reduce hypo-glycaemic unawareness events^2–5^. A major obstacle in intra-portal PITx is the devastating effect of the innate immune system, including the instant blood mediated inflammatory reaction (IBMIR). Islets express tissue factor that upon contact with blood activate coagulation factor VII which initiates the coagulation cascade, with platelet aggregation and clot formation around the islets. Islets in contact with blood also activates the complement cascade which potentiates the inflammatory reaction towards the islet graft^6^. These reactions are believed to cause the immediate loss of more than half of the transplanted islets^7^ and may also increase the risk of islet rejection later through adaptive immune response^8–10^. The importance of inhibiting the initial inflammatory/innate reactions has been increasingly recognized.

During the last decades, the non-hematopoietic effects of erythropoietin (EPO), including its anti-inflammatory and tissue protective mechanisms have been discovered^11^. Tissue under stress secretes higher concentration of EPO, that is required to activate a heterodimeric receptor of a combination of an EPO receptor (EPOR) and common ß-subunit, also called the innate repair receptor (IRR)^12^. Activation of IRR switches a cells energy expenditure to a cell protective pathway, which includes reduced activity of NK-kB and attenuation of pro-inflammatory cytokines^13^. These mechanisms contrast with the conventional hematopoietic properties of EPO following binding to the homodimeric EPOR.

The anti-inflammatory and cell protective properties of EPO have been shown to be cytoprotective in several organ systems during metabolic stress^14–17^. Using EPO or recombinant alternatives in nonhematopoietic indications in a clinical setting is however controversial. The increased hemoglobinemia, increment in platelet concentration and increased coagulation activity leads to hyperviscocity and possess an increased risk of thromboembolic complications^18^.

To harness EPOs beneficial effects without the risk of increased thromboembolism, the isolated structure of helix B of EPO, which selectively binds to and activates the IRR, was engineered as an 11 amino acid peptide and named cibinetide^19,20^.

Previously cibinetide has been evaluated in rodent intra-portal PITx models and has shown to improve the short-term islet function by reducing the postoperative inflammation in the transplantation site and subsequently reducing the adaptive immune responses, significantly improving long-term allograft survival in allogenic PITx^21,22^. These encouraging results suggest cibinetide as a potent addition to the immunosuppressive treatment arsenal also in clinical PITx. There are however important issues that remain to be clarified such as the IBMIR reaction, involving platelet activity and the coagulation pathways. Additionally, the previous beneficial effects of cibinetide were only studied in rodent models. The interspecies difference of islet physiology and architecture^23,24^ may play a role in the translation of the effect of cibinetide from rodent PITx to human PITx, as this has been shown to be difficult in the past^25,26^. This makes it important to apply a stepwise approach to clinical application. In preparation, we’ve moved from syngenic^21^ to allogenic^22^ and now to human islet transplantation models.

Herein we evaluate cibinetide’s possible protective effect on isolated human pancreatic islets in an inflammatory environment in in vitro models and in transplantation to athymic mice, characterizing its effect on the IBMIR reaction and graft function early after transplantation.

## Methods

### Ethical Considerations

The use of human islets and blood from healthy volunteers for this study was approved by the national Ethical Committee (etikprovningsmyndigheten) (2015/715-31/1) and (2016/1883-31/1) and performed in accordance with the principles of the Declaration of Helsinki. The human islet to NMRI mice PITx experiments was approved by the Swedish board of agriculture (number: S30-15 and 78-15).

### Human Pancreatic Islets

Human pancreatic islets from deceased donors were prepared and provided by Rudbeckslaboratoriet (Uppsala University, Sweden), as preciously described^27^. The isolated islets were transported and maintained in culture medium; Connaught Medical Research Labs 1066 media (CMRL1066, Corning, Manassas, VA, USA) supplemented with 10 mM HEPES (Invitrogen AB, Stockholm, Sweden), 10 mM nicotinamide (Swedish Pharmacy, Umeå, Sweden), 2 mM L-glutamine (Invitrogen), 50 μg/mL gentamicin (Invitrogen), 5 mM sodium pyruvate (Swedish Pharmacy, Umeå, Sweden), 20 μg/mL ciprofloxacin (Bayer, Leverkusen, Germany), and 10% human serum (Uppsala Blood Bank, Uppsala Sweden) in a humidified 5% CO_2_ atmosphere at 27°C until use.

### Animals

NMRI athymic mice were provided from Charles River, laboratories (Sulzfeld, Germany). The mice were maintained in a pathogen-free facility at Karolinska Institute, Stockholm, Sweden. The study was conducted according to the Guidelines for the Use of Laboratory Animals of Karolinska Institute.

### Reagents and Recombinant Cytokines

Cibinetide was provided by Araim Pharmaceuticals, Inc. (Tarrytown, NY, USA). Cibinetide stock solution (1.2 mg/mL, 1 mmol/L) was dissolved in phosphate buffered saline (PBS), aliquoted and stored at -20°C until use. The doses of cibinetide (100 nmol/L in in vitro study and 120 µg/kg in in vivo model) were selected based on our previous studies^21,22^. Recombinant human interleukin (IL)-1β, tumor necrosis factor (TNF)-α, and interferon (IFN)-γ were purchased from PeproTech Inc., (Rocky Hill, NJ, USA).

### Pancreatic Islet Culture with Pro-Inflammatory Cytokines

Human pancreatic islets (20 islets) were handpicked under microscope and incubated in 24-well non-attaching culture plates (Sigma-Aldrich, Missouri, USA) with 0.5 mL islet culture medium at 37°C with 5% CO_2_ in a humidified atmosphere. Islets were incubated with or without human proinflammatory cytokines (IL-1β; 50 IU/mL, TNF-α; 1000 IU/mL, and IFN-γ; 1000 IU/mL) in the presence or absence of cibinetide (100 nmol/L) for 18 hours.

### ATP Content and Caspase 3/7 Activity

Pancreatic islets were collected after the co-culture with proinflammatory cytokines and put in a cell suspension (20 islets in 80 µL of Hanks Balanced Salt Solution (HBSS) (Karolinska University Hospital, Stockholm, Sweden)) and mixed thoroughly with the same volume of CellTiter-Glo® reagent or Caspase-Glo 3/7 reagent (Promega Corp., Madison, USA). Each sample was divided into duplicates (60 µl per well) in 96-well plates, half-area white flame (Corning, NY, USA), After 15 minutes (for CellTiter-Glo®) or 30 minutes (for Caspase-Glo 3/7) incubation, the luciferase activity was measured with luminometer (Biotek FLx800™ Multi-Detection Microplate Reader operated by Gen5™ Data Analysis Software) with an integration time of 1 second/well. The measured luminescence is proportional to ATP content or caspase 3/7 activities. To correct for the number of cells in each sample, the amount of double-stranded DNA (dsDNA) was measured by dyeing the sample with Quant-iT™ PicoGreen® dsDNA Assay Kit (Invitrogen, Carlsbad, USA) according to the manufacturer’s instructions. Fluorescence activity was measured with a fluorometer (Biotek FLx800™ Multi-Detection Microplate Reader operated by Gen5™ Data Analysis Software).

### Dynamic Glucose Perfusion Assay

Insulin production form isolated islets after in vitro culture with or without pro-inflammatory cytokines was evaluated by using glucose perfusion assays, as previously described^28^. After incubating for 18 hours, 20 islets were placed in filter-closed chambers and perfused sequentially for 126 min, with 1.67 mmol/L glucose for the first 36 min, 20 mmol/L for the next 42 min, and again with 1.67 mmol/L glucose for 48 min. The effluents were collected every four minutes. The insulin levels in the effluent were determined using human insulin enzyme-linked immune sorbent assay (ELISA) (Mabtech AB, Nacka, Sweden). Area under the curve (AUC) was calculated for the low and high glucose perfusion period.

### In Vitro IBMIR Model

IBMIR was examined using a modified tubing loop model to mimic the portal venous blood flow^6,29^. ABO-blood type identical human blood from healthy voulunteer was collected in a heparin coated Falcon tube within 15 minutes before the assay. Four mL blood was aliquoted into each to heparin coated polyvinyl chloride (PVC) tube (20 cm long, inner diameter 6 mm; Corline Biomedical, Uppsala, Sweden). Human pancreatic islets were washed once with HBSS and were injected into each tube in a pellet form (3 µL). The PVC loops were rotated (22 rpm) for 60 minutes at 37°C. Drugs (heparin and cibinetide) was added into the tubes according to the groups; (1) only blood, (2) islets (3) islets with heparin (0.4 U/mL blood), (4) islets with heparin and cibineitde (100 nmol/L). After 60 minutes rotation, 32 µL of 0.5 M EDTA (the final concentration 4 mM/mL) was injected into each loop to halt the coagulation cascade. From each tube, 300 µL of blood was collected and various blood cells were counted by using cell spectrometer KX-21 N (Sysmex, Kobe, Japan). The remaining blood was centrifuged at 300 g for 10 min, and the supernatant was further centrifuged at 2500 g for 15 min at 4°C to separate plasma and platelets. The plasma was collected and stored at –80°C for further analysis. The high mobility group box-1 protein (HMGB-1), complement component 3a (C3a) factor activation and thrombin-anti thrombin complex (TAT) ELISA kits were perchased from: BioSite (Täby, Sweden), eBioscience (C3a, Vienna, Austria), Abcam (TAT, Cambridge, UK).

### Human Pancreatic Islet Transplantation to Athymic Mice

Isolated human pancreatic islets (1250 IEQ) were transplanted into the liver of non-diabetic athymic mice (NMRI) via the portal vein as previously described^21^. Cibinetide (120 µg/kg) was administrated intraperitoneally (IP) just before and at 0, 6, and 24 hours after PITx based on our previous study^21^. In the previous clinical evaluations performed both in normal volunteers and subjects with painful small fiber neuropathy, subcutaneous doses up to 8 mg/dose were shown to be safe, with ≥4 mg being effective^30–32^. Based on formal pharmacokinetic studies, a 120 µg/kg dose of cibinetide administered subcutaneously to the rat or New Zealand white rabbit produced a peak area under the curve values which were comparable to those obtained from humans administered 6 mg (∼90 µg/kg) of cibinetide (rat = 275 ng·min/mL; rabbit=284 ng·min/mL; human=245 ng·min/mL; Araim Pharmaceuticals, unpublished data). Vehicle (PBS) was administered to control group animals. The livers and serum samples were obtained from transplanted animals at 6 days after transplantation.

### Human Insulin Content in the Livers After islet Transplantation

The extraction method used was described by Jackson Laboratories (https://www.diacomp.org/shared/document.aspx?id=73&docType=Protocol). The whole livers were extirpated during euthanasia and placed into 10 ml acid-ethanol (1.5% HCl and 70% ethanol) and incubated at -20°C for 20 hours before and after homogenization. The tissue tubes were centrifuged at 3000 rpm for 15 minutes at 4°C and the supernatant was collected. Human insulin amounts were measured using ELISA kit (Invitrogen, CA, USA). The liver from a non-transplanted mouse was used as negative control and proved to be negative of human insulin.

### Human C-Peptide in Mouse Serum

The blood was collected from transplanted NMRI animals at the time of euthanasia and centrifuged at 3000 g for 10 minutes. The serum was frozen at -80°C until use. Human C-peptide was measured by ELISA (Mercodia, Uppsala, Sweden).

### Immunofluorescence Microscopy

The livers obtained from each group of animals were covered in OCT compound (Sigma-Aldrich) and frozen in liquid nitrogen. Approximately 200 slices of liver were sectioned in each group (5 µm thick/slice) and a total of 22 insulin positive locations were identified. The sections were fixed with 4% formaldehyde in PBS, washed, and blocked with 5% donkey serum in PBS with 0.05% Tween20 for 30 min at room temperature (RT). The sections were incubated overnight at 4°C with mouse IgG anti mouse CD11b (1/50 diluted, eBioscience) a in humidified atmosphere. The sections were washed with PBS contained 0.05% Tween (washing buffer) and incubated with FITC goat anti mouse IgG (1/100 diluted, Jackson ImmunoResearch, Cambridge, UK) for 60 minutes at RT. After the incubation, the sections were washed and incubated with rhodamine-conjugated anti-insulin antibodies (1/50 diluted, Mabtech AB) for 60 minutes at RT. After washing, the sections were incubated with DAPI (10 times diluted stock solution, ThermoFisher Scientific) for 1 minutes and rinsed with PBS then mounted with fluorescence mounting buffer. Insulin staining was observed by fluorescence microscope (Olympus Ix70 Inverted Fluorescence & Phase Contrast Tissue Culture Microscope, Olympus, Solna, Sweden). The tissue slices immunofluorescence imaging (2D and 3D) and imaging analysis was performed at the Live Cell Imaging facility, Karolinska Institute, Sweden. First, the complete surface 2D image of the tissue slices was acquired to identify the insulin-positive integrated islets in the section. Second, image stacks of all the identified islets were acquired, using the spinning disk confocal mode (Nikon Eclipse Ti microscope), in order to assemble the 3D images of the islets within liver tissue slices, with image sharpness quality comparable to confocal. Imaris v.8 software (Bitplane, Oxford instruments) was used to identify insulin-positive areas and CD11b^+^ cells with the reconstructed 3D image for the slices and to quantify the number of CD11b^+^ cells in proximity (50 μ) to insulin-positive areas for the acquired 3D reconstructions. The CD11b^+^ infiltrating cell/insulin positive area ratio was then calculated.

### Statistical Analysis

Wilcoxon’s test was used when comparing two paired groups with one outcome variable. One-way ANOVA was used, when comparing >2 groups with one outcome variable, Two-way ANOVA, Tukey’s multiple comparisons test were used when comparing >2 categories of outcome variables. Mann-Whitney U-test was used when comparing two non-paired groups with one outcome variable groups of. A *p*-value <0.05 was considered statistically significant. All calculations were performed using GraphPad Prism® version 8 (GraphPad Software Inc., San Diego, CA, USA).

## Results

### Cibinetide Maintained Human Islet Viability in a Pro-Inflammatory Milieu

**Figure 1.**
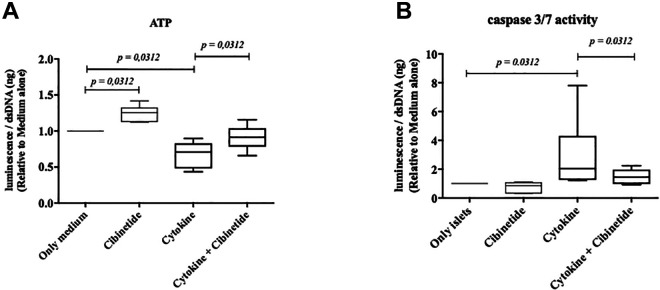
ATP content **(A)** and caspase 3/7 activity **(B)** of 20 human islets cultured in islet medium with or without addition of a pro-inflammatory cytokine cocktail (IL-1β; 50 IU/mL, TNF-α; 1000 IU/mL, and IFN-γ; 1000 IU/mL) in the presence or absence of 100 nmol/L cibinetide for 18 hours. The values are depicted as the lower quartile, median, and upper quartile (boxes), lower and upper whiskers represent minimum and maximum ranges. When cytokines were added to the islet culture, the ATP levels was reduced and capsase 3/7 was elevated. When cibinetide was present in the culture with islets and cytokines, the ATP levels were significant higher (Wilcoxon test *p =* 0.0312) while caspase 3/7 decreased (Wilcoxon test *p =* 0.0312) compared to the culture with cytokines and islets (*n* = 6). In the culture with islets and medium without the pro-inflammatory cytokine cocktail, the ATP levels were also significant higher compared the control group without cibinetide (Wilcoxon test *p = 0.*0312).

To evaluate cibinetides islet protective efficacy, human islets were cultured in medium with a mixture of pro-inflammatory cytokines for 18 hours. Islets cultured in pro-inflammatory cytokines showed a diminished ATP and increased caspase 3/7 activity. When cibinetide was present in the culture, the ATP levels were maintained (Wilcoxon test *p =* 0.0312) while caspase 3/7 was significant lower (Wilcoxon test *p =* 0.0312) compared to the culture with only cytokines and islets (Fig. 1). In the culture with islets and medium without pro-inflammatory cytokines, the ATP levels were also significant higher compared the control group without cibinetide (Wilcoxon test *p =* 0.0312).

### Cibinetide Maintains the Insulin Secreting Function of Human Islets Following pro-Inflammatory Exposure

The insulin secreting capacity of islets cultured in pro-inflammatory cytokines for 18 hours with or without cibinetide treatment was evaluated in dynamic glucose perfusion assay.

**Figure 2.**
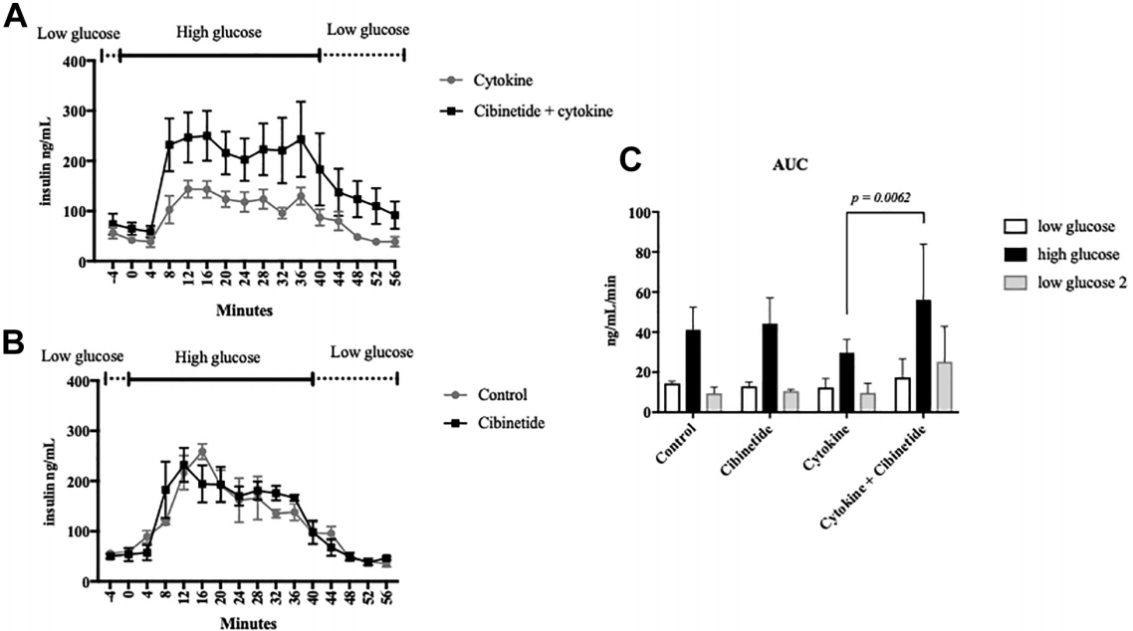
Dynamic glucose perfusion of islets after 18 hours incubation with pro-inflammatory cytokines with or without treatment of 100 nmol cibinetide **(A)** and islets cultured in medium without cytokines **(B)**. Islets were perfused with solution with low glucose (1.67 mM) from -4 to 0 minutes, and thereafter perfused with high glucose solution (20 mM) for 40 minutes followed by low glucose (1.67 mM). Human insulin was analyzed from the perfusate collected every 4 minutes using ELISA. Cibinetide treatment had no effect in insulin secreting capacity in the islets cultured in medium (*n* = 4) **(B)**, while cibinetides improved the insulin secretion in response to a high glucose concentration in the islets that were cultured in pro-inflammatory cytokines (*n* = 5) **(A)**. There was significant increased insulin secretion in response to high glucose in the islets that were cultured in pro-inflammatory cytokines and cibinetide treatment compared to those cultures maintained in pro-inflammatory cytokines alone (Two-way ANOVA, Tukey’s multiple comparisons test *p =* 0.0062 **(C)**.

Islets exposed to pro-inflammatory cytokines showed poor insulin secretion in response to a perfusate with a high glucose concentration. In contrast, islets that were cultured with cytokines and cibinetide had a significantly higher insulin secretion (area under the curve; AUC) when stimulated with high glucose (Tukey’s multiple comparison test *p =* 0.0063) (Fig. 2A, C). Islets that were cultured in medium without pro-inflammatory cytokines were also subjected to dynamic glucose perfusion. However, cibinetide alone did not improve the insulin secreting capacity of these islets to high glucose (Fig. 2B).

### Cibinetide Reduce the Platelet Consumption in the in vitro IBMIR Model

**Figure 3.**
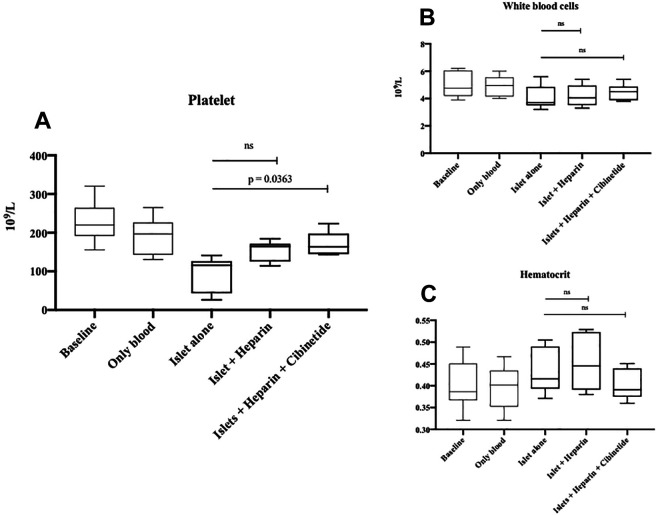
Platelets, white blood cells and hematocrit levels in whole blood after 60 minutes perfusion through circular heparinized PVC tubes containing islets. Baseline represents the start value directly after blood donation from a healthy volunteer. The values are depicted as the lower quartile, median, and upper quartile (boxes), lower and upper whiskers represent minimum and maximum ranges. Cibinetide and heparin treatment in the loops with islets significantly reduced the IBMIR related platelet consumption (One-way ANOVA, Tukey’s multiple comparisons test *p =* 0.0363). This effect is not seen when only heparin was used **(A)**. There was no difference in white blood cell count **(B)** or hematocrit in the different treatments **(C)** (*n* = 6).

During the 60 minutes rotation of the PVC loops, the platelet counts were decreased. When heparin (0.4 IU/ml of blood) and cibinetide (100 nmol/L) was added into the PVC loops, the thrombocyte levels were significantly higher (*p* = .0363; Tukey’s Multiple comparison) (Fig. 3A). This effect was not seen when only heparin was added to the PVC loops. The hematocrit and white blood cells were not affected by either heparin or cibinetide (Fig. 3B, C). Cibinetide had no effect on C3a activation or HMGB-1 levels in isolated plasma from the PVC loops (data not shown). There was no difference in TAT levels between the groups (data not shown).

### Treatment with Cibinetide Improves Islet Engraftment of Human Islets into the Livers of NMRI Mice After PITx

**Figure 4.**
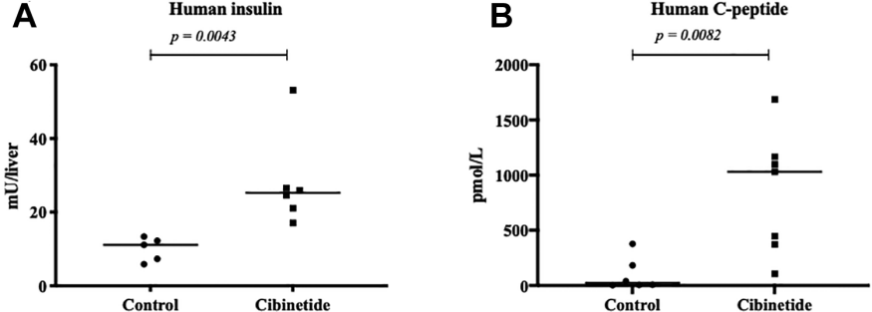
Human insulin content per liver **(A)** and human C-peptide levels in serum **(B)** of NMRI mice that were transplanted with 1250 EQ human islets and sacrificed 6 days after transplantation. Individual values are shown in plot, black line represents mean value. There was higher amount of human insulin per liver in cibinetide treated mice (*n* = 6) compared with the control group mice (*n* = 5) (Mann Whitney U-test *p =* 0.0043) **(A)**. Concordantly, the cibinetide treated recipients (*n* = 7) had higher amount of human C-peptide in the serum compared to the control group (*n* = 6) at 6 days after PITx (Mann Whitney U-test *p = 0.*0082) **(B)**.

At six days after human islet to NMRI mice PITx, the cibinetide treated group showed significantly higher amount of human insulin in the liver compared to the control group (*p* = .0043; Mann Whitney U-test, Fig. 4A). Concordantly, the cibinetide treated NMRI mice showed higher human C-peptide in the serum compared to the control group (*p* = .0082; Mann Whitney U-test, Fig. 4B).

### Cibinetide Treatment Reduces the Numbers of CD11b^+^ cells Infiltrating Transplanted Human islets in the Livers of NMRI Mice

**Figure 5.**
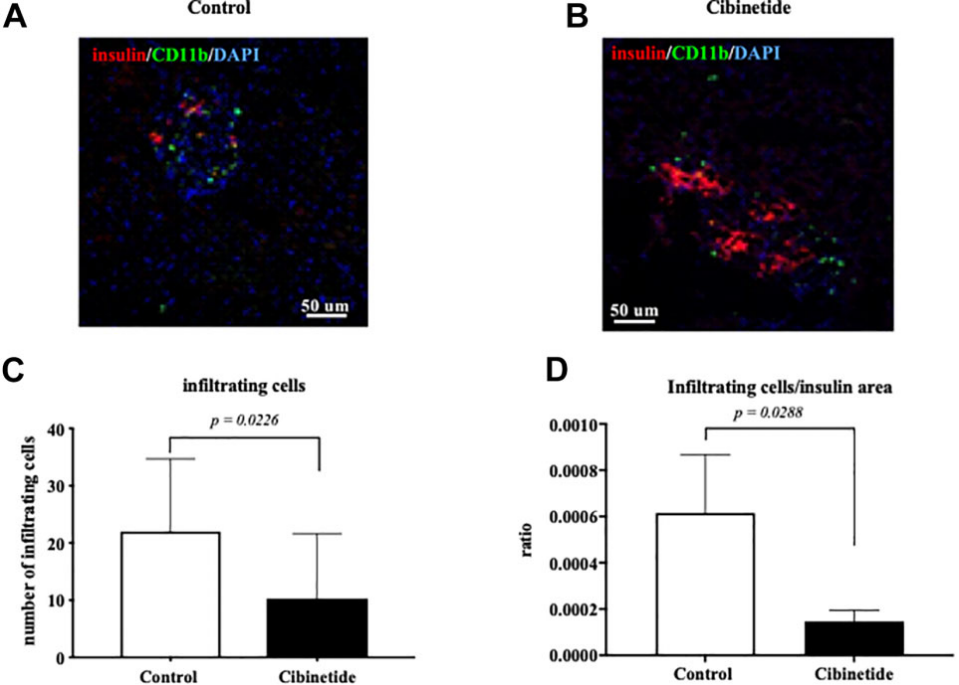
Histological evaluation of livers from NMRI mice extirpated 6 days after PITx with human islets. Of the screened sections, 22 sections were engrafted human islets were evaluated with anti- insulin and anti-CD11b staining. Insulin staining in red, cell nucleus (DAPI) in blue and CD11b in green. The 3D images of all the identified islets were acquired, using the spinning disc confocal mode on the Nicon Eclipse Ti microscope. An example of a liver section from the control group showing an insulin positive area with CD11b infiltration around and into the islets grafts (A), and another liver section from the cibinetide treated group shows lesser infiltration of CD11b in relation to insulin positive islets (B). Imaris v.8 software from Bitplane, Oxford instruments was used to identify the insulin-positive area, CD11b+ cells and quantify the number of CD11b+ cells in proximity (50 μ) from the insulin-positive area for the acquired 3D images of the islets within liver sections. Of the examined sections, the cibinetide treated mice had significant lower infiltrating cells around islets grafts (Mann Whitney *p* = 0.0226) (C). Numbers of CD11b+ cell infiltration / insulin positive areas in the liver sections of cibinetide treated mice were also significantly lower compared to the control group (Mann Whitney U-test *p* = 0.0288) (D).

Histological liver sectioning (Fig. 5A, B) revealed that cibinetide treated mice had a lower number of infiltrating CD11b^+^ cells around insulin positive area compared to the control group (Mann Whitney U-test *p = 0.*0226) (Fig. 5C). The CD11b^+^ infiltrating cells to insulin positive area ratio was significantly lower in the cibinetide group compared to the control group (Mann Whitney U-test *p =* 0.0288) (Fig. 5D).

## Discussion

In this study, we demonstrate cibinetide’s beneficial effects on human islets isolated from deceased donors in the aspects of PITx. Cibinetide treatment maintained human islets stability and insulin producing function in a pro-inflammatory milieu. In an in vitro portal flow mimicking model, cibinetide treatment showed a reduction of IBMIR related thrombocyte consumption. In vivo human islet to NMRI mice intra-portal PITx showed increased human C-peptide levels in the mice serum and increased insulin amount in the recipient livers from animals receiving cibinetide. Histological evaluation of liver grafts from NMRI mice recipients transplanted with human islets also showed fewer infiltrating CD11b^+^ cells around the islet grafts when cibinetide was used.

The effects of cibinetide in PITx are pleiotropic. One mechanism is its cytoprotective property directly affecting islets during inflammation. The physiology of brain death of the donor, islet isolation process and the transplantation procedure in the recipient all create a pro-inflammatory milieu. Islets residing in an environment with inflammatory cytokines undergo damage and apoptosis^33,34^. Subjecting isolated human islets in culture with pro-inflammatory cytokines reduced their ATP content and increased caspase activity, while these were maintained when islets were treated with cibinetide. This has previously been shown in rodent islets^21,22^, and the effect is mainly believed to be caused by cibinitide’s direct effect on the cells within the islets. The components of IRR (EPO-R and CD131) are generally intracellular in the non-inflammatory state, while the receptors translocate to the cell surface only in stressful conditions^13^. Occupancy of the IRR activates the intra cellular JAK-STAT-Bcl2 pathways that induce anti-apoptotic pathways and inhibits apoptotic^11^, which switches cells energy expenditure to survival. In this present study, we confirm this effect by the reduced caspase levels and maintained ATP content also in human islets in the pro-inflammatory cytokine culture. Interestingly, the ATP levels of isolated islets in a culture without pro-inflammatory cytokines also showed higher values when cibinetide was administered in the culture. This could be explained by the fact that isolated islets were already traumatized by the mechanical injuries of the isolation process and ischemia. The benefit of this mechanism is also shown by the significantly improved glucose sensitivity and insulin secreting ability of the cibinetide treated human islets after cytokine exposure compared to the control group (Fig. 2A).

Secondly it is important before clinical application, to clarify the difference of using a selective IRR agonist compared to EPO. Conventional EPO leads to an activation of blood coagulation, platelet hyperactivity, hypertension and hyper viscosity after activation of the EPO-R^18^, and has been clinically associated with an increased risk of thromboembolism^35–37^, especially in non-anemic patients. The typical patient undergoing islet transplantation is type-1 diabetics without renal failure, hence generally has a normal coagulation and hemoglobin parameters. The selective IRR agonist cibinetide however mechanistically only agonize the IRR receptor without any earlier evidence of activity on the EPO-R.

Considering coagulation is one of the major pathophysiologic mechanisms underlying IBMIR, which leads to a severe initial islet loss, it is of importance before clinical implementation to characterize if there is any risk of spillover effect of conventional EPO-R that can aggravate the IBMIR reaction. This has previously has not been investigated. Our present results show that cibinetide does not lead to an aggravation of IBMIR. On the contrary, the reduced platelet consumption in the in vitro IBMIR model when heparin and cibinetide was administered in the loops indicates a reduced IBMIR related clot formation, which can be seen as possible second mechanism. Cibinetide did however not affect the C3a levels during the one-hour exposure to in vitro model of portal circulation. Even though coagulation, complement activity, infiltration and aggregation of platelets and leukocytes around transplanted islets synergistically orchestrates IBMIR, they are parallel pathways. This may indicate that cibinetide effects targets within IBMIR other than complement in the short term. In this study we did not find any difference of HMGB-1 levels in the plasma between the cibinetide and control group in the loop model. Previous reports indicated that it took 3-6 hours after stimuli (depending on the experimental models) before an increase of HMGB-1 was detected^38^. Due to the fact that the HMGB-1 levels in this present study were analyzed in the plasma collected directly after the 1-hour rotation in the in vitro IBMIR model, it may be a to short period to detect differences in HMGB-1 levels between the groups.

A third mechanism towards a more successful transplantation is cibinetides attenuating effect on the recipient’s innate immune cells. Several immune cells express EPOR, e.g. macrophages, T-cells, dendritic cells^39,40^. While we previously reported that cibinetide could directly affect dendritic cells maturation^22^ and macrophage activity^21^ in rodent models, mechanistically it has also been shown that IRR activation reduces the binding affinity of NFκB to DNA^41^, which at a transcriptional level leads to reduced production of inflammatory chemokines and cytokines such as MCP-1, TNF-α and IL-6^42,43^. This consequently may lead to reduced infiltration of innate immune cells such as macrophages and other immune cells to the transplant site. In the present study, this concept is strengthened when examining the histological liver sections from NMRI mice that were transplanted with human islets. It showed significantly reduced pro-inflammatory CD11b^+^ cell infiltration around insulin positive areas in cibinetide treated animals compared to the control group, indicating reduced infiltration of innate immune cells around the transplanted islets.

We suggest that all of the above discussed mechanisms of cibinetide improve the outcome of PITx. In the present study, these synergistic protective mechanisms result in the significantly higher levels of serum human C-peptide and human insulin in the livers of cibinetide treated NMRI mice after PITx with human islets compared to the control group, indicating an overall improved engraftment and function of the transplanted islets short term after transplantation. A higher number of survived engrafted islets is also expected to translate into an improved long-term outcome, which we previously demonstrated in an allogenic mouse PITx model^22^, but needs to be further investigated in a clinical setting. Beside these encouraging effects, our previous report suggests that the effect on the innate immunity will lead to reduced allo-reactivity^22^, this matter is not investigated further in this report. However, since cibinetide mainly targets the innate immunity, the beneficial effects may not be limited to allogenic PITx but may also benefit in the instances of autologous PITx after non-malignant indications for total pancreatectomy.

Cibinetide has been evaluated in several clinical trials showing a favorable safety profile^31,32,44–46^ and clinical benefit in several clinical settings, e.g. ability to reduce neuropathy in sarcoidosis and type 2 diabetes.

In summary, the result from this study confirms cibinetide’s anti-inflammatory and cell protective effects on isolated human islets in the inflammatory environment. In the perspective of PITx, cibinetide has shown several beneficial mechanisms that may improve the outcomes. The potency of this promising drug should be evaluated in the clinical PITx setting.

## Supplemental Material

Supplemental Material, sj-docx-1-cll-10.1177_09636897211039739 - Cibinetide Protects Isolated Human Islets in a Stressful Environment and Improves Engraftment in the Perspective of Intra Portal Islet Transplantation Click here for additional data file. Supplemental Material, sj-docx-1-cll-10.1177_09636897211039739 for Cibinetide Protects Isolated Human Islets in a Stressful Environment and Improves Engraftment in the Perspective of Intra Portal Islet Transplantation by Ming Yao, Anna Domogatskaya, Nils Ågren1, Masaaki Watanabe, Kazuaki Tokodai, Michael Brines, Anthony Cerami, Bo-Göran Ericzon, Makiko Kumagai-Braesch and Torbjörn Lundgren in Cell Transplantation

## Abbreviations

ATP: Adenosine triphosphate
AUC: Area under the curve
C3a: Complement Component 3a
CMRL: Connaught Medical Research Labs
CD: cluster differentiation
DAPI: 4,6-diamidino-2-phenylindole
dsDNA: Double-stranded DNA
EDTA: Ethylenediaminetetraacetic acid
ELISA: Enzyme-linked immune sorbent assay
EPO: Erythropoietin
EPOR: EPO receptor
HBSS: Hanks Balanced Salt Solution
HEPES: N-(2-Hydroxyethyl) piperazine-N′-(2-ethanesulfonic acid)
HMGB1: high mobility group box-1
IRR: Innate repair receptor
IBMIR: Instant blood mediated inflammatory reaction
IFN: Interferon
IL: Interleukin
NMRI: Naval Medical Research Institute
PITx: Pancreatic islet transplantation
PBS: Phosphate buffered saline
PVC: Polyvinyl chloride
RT: Room temperature
TAT: Thrombin-anti thrombin complex
TNF: Tumor necrosis factor
