# Supplementary material for: Cibinetide Protects Isolated Human Islets in a Stressful Environment and Improves Engraftment in the Perspective of Intra Portal Islet Transplantation
Source: Cell Transplant. 2021 Sep 9;30:09636897211039739. doi: 10.1177/09636897211039739 (PMC8436319; doi:10.1177/09636897211039739)
Supplement: Supplemental Material, sj-docx-1-cll-10.1177_09636897211039739 - Cibinetide Protects Isolated Human Islets in a Stressful Environment and Improves Engraftment in the Perspective of Intra Portal Islet Transplantation [file sj-docx-1-cll-10.1177_09636897211039739.docx]

# Author response to reviewers

#### MANUSCRIPT ID: CT-3218

#### MANUSCRIPT TITLE: Cibinetide protects isolated human islets in a stressful environment and improves engraftment in the perspective of intra portal islets transplantation

| REVIEWER 1 COMMENT | AUTHOR RESPONSE | PAGE NUMBER |
| --- | --- | --- |
| 1. Although platelet consumption is reduced in the in vitro loop model of IBMIR with cibinetide treatment, HMGB1 and complement are no different. Can the authors speculate on why they did not see evidence for benefit in these inflammatory and complement markers in this model? | Thank you for this important notice.  The coagulation cascade, complement activation, platelets and leucocytes all together orchestrate the devastating IBMIR. Tissue factor on islets cell surfaces is upregulated by the contact with blood which lead to the activation of the coagulation cascade. In parallel the complement system is activated. Activation of these processes leads to recruitment of leucocytes and consumes platelets as thrombi is formed encapsulating islets.  Even though all these systems are connected, inhibition of single point(s) of these reactions may reduce the magnitude of the final effect. In this study, we see a reduction of platelet consumption with the addition of cibinetide in our in-vitro IBMIR model. However, the complement C3a levels are unchanged. This may indicate that cibinetides effect other targets within IBMIR than the complement cascade to the point of C3a.  The exact mechanistic target point of cibinetide within IBMIR reaction is yet to be elucidated.  The section below is added to the discussion page: 13-14  *“Cibinetide did however not affect the C3a levels during the one-hour exposure to in-vitro model of portal circulation. Even though coagulation, complement activity, infiltration and aggregation of platelets and leukocytes around transplanted islets synergistically orchestrates IBMIR, they are parallel pathways. This may indicate that cibinetide effects targets within IBMIR other than complement in the short term. “*  In this study we did not see any difference of HMGB-1 levels in the plasma collected directly after the in-vitro IBMIR model with or without addition of cibinetide in the loops.  In a previous study, mouse islets were cultured with or without proinflammatory cytokines showed higher levels of HMGB-1 in the cytokine group after 6 hours, and HMGB-1 peak at 24 hours. In the same study, histological findings suggest that HMGB-1 was present in the cytoplasm 3 hours after PITx, while it was only present within the nucleus of naïve pancreatic islets ^1^. In another study, HMGB-1 was measured in the serum of mouse PITx recipients 12 hours after transplantation with or without the use of a NF-κB blocker ^2^. Due to the fact that the HMGB-1 levels were analyzed in the plasma collected directly after the 1-hour rotation in the in-vitro IBMIR model, it may in this case be to short period to detect a difference of HMGB-1 levels between the groups.  The section below is added to the discussion at page: 14  *“In this study we did not find any difference of HMGB-1 levels in the plasma between the cibinetide and control group in the loop model. Previous reports indicated that it took 3-6 hours after stimuli (depending on the experimental models) before an increase of HMGB-1 was detected* *^1^. Due to the fact that the HMGB-1 levels in this present study were analyzed in the plasma collected directly after the 1-hour rotation in the in-vitro IBMIR model, it may be a too short period to detect differences in HMGB-1 levels between the groups. “* | 13, 14 |
| 2. The animal studies are short-term (6 days).  One would expect that an immediate protection against IBMIR will translate into a long-term effect but it may be worthy of further mention in the discussion. | Thank you very much for pointing out this important issue. We agree that the expected long-term benefit is a very important aspect to address.  In this study, cibinetide reduced damage caused by inflammation and innate immune responses.  In terms of the multifaceted IBMIR reaction, which could cause the loss of more than half of the transplanted islets, we show that cibinetide reduced platelet consumption as discussed in the comment nr 1. Based on these results the anticipated effect should also result in a long-term beneficial effect in the overall transplantation outcome.  This was indeed shown in our previous allogenic mouse PITx model using cibinetide, where cibinetide in combination with tacrolimus administration improved the long-term outcome when compared to only tacrolimus treatment^3^.  This important consideration is added to the discussion section on page: 14    *“In the present study, these synergistic protective mechanisms result in the significantly higher levels of serum~~-~~human-C-peptide and human insulin in the livers of cibinetide treated NMRI mice after PITx with human islets compared to the control group, indicating an overall improved engraftment and function of the transplanted islets short term after transplantation. A higher number of survived engrafted islets is also expected to translate into an improved long-term outcome, which we previously demonstrated in an allogenic mouse PITx model^3^, but needs to be further investigated in a clinical setting. “* | 14 |
| 3. How do the doses used in this study translate into an equivalent human dose?  Is the dosing similar to what has so far been shown to be safe in the clinical trials referenced in the discussion on page 16 lines 52-55? | Thank you for this question.  In the previous clinical evaluations performed both in normal volunteers and subjects with painful small fiber neuropathy, subcutaneous doses up to 8 mg / dose were shown to be safe, with ≥4mg being effective^4-6^. Based on formal pharmacokinetic studies, a 120 µg/kg dose of cibinetide administered subcutaneously to the rat or New Zealand white rabbit produced a peak area under the curve values which were comparable to those obtained from humans administered 6 mg (~90 µg/kg) of cibinetide (rat=275 ng·min/mL; rabbit=284 ng·min/mL; human=245 ng·min/mL; Araim Pharmaceuticals, unpublished data).  This section above is also added in the method part of the manuscript on page: 8-9 | 8-9 |
| 4. Note that heparin is spelled at various points in the methods and figures as heparine. | Thank you for noticing this. The misspellings of heparin are corrected throughout the manuscript. |  |

**References**

1.	Matsuoka N, Itoh T, Watarai H, Sekine-Kondo E, Nagata N, Okamoto K, Mera T, Yamamoto H, Yamada S, Maruyama I and others. High-mobility group box 1 is involved in the initial events of early loss of transplanted islets in mice. J Clin Invest 2010;120(3):735-43.

2.	Kuraya D, Watanabe M, Koshizuka Y, Ogura M, Yoshida T, Asahi Y, Kamachi H, Nakamura T, Harashima H, Ozaki M and others. Efficacy of DHMEQ, a NF-kappaB inhibitor, in islet transplantation: I. HMGB1 suppression by DHMEQ prevents early islet graft damage. Transplantation 2013;96(5):445-53.

3.	Yao M, Watanabe M, Sun S, Tokodai K, Cerami A, Brines M, Ostenson CG, Ericzon BG, Lundgren T, Kumagai-Braesch M. Improvement of Islet Allograft Function Using Cibinetide, an Innate Repair Receptor Ligand. Transplantation 2020;104(10):2048-2058.

4.	Brines M, Dunne AN, van Velzen M, Proto PL, Ostenson CG, Kirk RI, Petropoulos IN, Javed S, Malik RA, Cerami A and others. ARA 290, a nonerythropoietic peptide engineered from erythropoietin, improves metabolic control and neuropathic symptoms in patients with type 2 diabetes. Mol Med 2015;20:658-66.

5.	Culver DA, Dahan A, Bajorunas D, Jeziorska M, van Velzen M, Aarts L, Tavee J, Tannemaat MR, Dunne AN, Kirk RI and others. Cibinetide Improves Corneal Nerve Fiber Abundance in Patients With Sarcoidosis-Associated Small Nerve Fiber Loss and Neuropathic Pain. Invest Ophthalmol Vis Sci 2017;58(6):BIO52-BIO60.

6.	Heij L, Niesters M, Swartjes M, Hoitsma E, Drent M, Dunne A, Grutters JC, Vogels O, Brines M, Cerami A and others. Safety and efficacy of ARA 290 in sarcoidosis patients with symptoms of small fiber neuropathy: a randomized, double-blind pilot study. Mol Med 2012;18:1430-6.
